# Frequency stabilization of multiple lasers to a reference atomic transition of Rb

**DOI:** 10.1038/s41598-022-24952-6

**Published:** 2022-11-30

**Authors:** Shubham Utreja, Harish Rathore, Manoj Das, Subhasis Panja

**Affiliations:** 1 CSIR-National Physical Laboratory, Dr. K. S. Krishnan Marg, New Delhi, 110012 India; 2 Academy of Scientific and Innovative Research (AcSIR), Ghaziabad, 201002 India

**Keywords:** Optics and photonics, Optical physics, Optical techniques, Atomic and molecular physics

## Abstract

Modern atomic clocks based on the interrogation of an atomic transitions in the optical regions require multiple lasers at different wavelength for producing atomic ions, trapping and laser cooling of neutral atoms or atomic ions. In order to achieve highest efficiency for laser cooling or any other atomic transition, frequencies of each of the lasers involved need to be stabilized by mitigating its drifts or fluctuations arise due to ambient temperature variation or other kind of perturbations. The present article describes simultaneous frequency stabilization of multiple number of lasers, required for production and laser cooling of ytterbium (^171^Yb) ions, to a reference transition frequency of rubidium (Rb) atoms. In this technique, a diode laser operating at ~ 780 nm is frequency stabilized to one of the Doppler broadening-free absorption peak of rubidium atoms (^85^Rb) and then used as a reference frequency for calibrating a wavelength meter and subsequent simultaneous frequency stabilization of four lasers operating at different wavelengths.

## Introduction

Frequency stabilization of lasers is a prerequisite for all kind of experiments related to laser cooling of atoms or atomic ions. Laser frequency stabilization or frequency locking is also an essential requirement for precision experiments^1–4^, e.g., optical frequency standards or optical clock based on the interrogation of ultra-narrow atomic transitions, which requires multiple number of stable laser frequencies. Optical atomic clock, realized through interrogation of a quadrupole transition {4f^14^ 6 s 2S_1/2_|F = 0, m_F_ = 0 > →4f^14^5D 2D_3/2_|F = 2, m_F_ = 0 >} of a single trapped ytterbium ion (^171^Yb^+^) ion at 435 nm, requires as many as four narrow linewidth(~ 23 MHz)^5^ and stable lasers at wavelengths around 399 nm, 369.5 nm, 935 nm and 760 nm respectively for probing transitions related to photoionization, laser cooling^6,7^ of ytterbium (^171^Yb^+^) ions and repumping of the metastable states of the ions^5,8,9^. Such a state-of-the art experiment demands accurate and precise regulation of all of its components, hence, all the required lasers need to be frequency stabilized so that desired transitions are probed accurately and effectively. Several techniques are being used for laser frequency locking or frequency stabilization, e.g., locking of laser’s output frequency to a highly stable Fabry-Parrot cavity using Pound–Drever–Hall technique^10,11^, another commonly used technique is locking of laser to a reference atomic transition frequency on its Doppler broadening free transition peak^12–14^ and now a days wavelength meters^15–17^ are also being used in different precision experiments. The choice of a locking technique depends on experimental demand for the level of accuracy. For example, precision spectroscopy or high accuracy metrology-related experiments demand relative frequency instabilities^18^ better than 10^−15^ whereas other experiments such as atomic spectroscopy or atom cooling can be performed with laser frequencies having short-term instabilities ≤ 10^−10^.

Laser cooled ytterbium ion (^171^Yb^+^) offers two ultra-narrow clock transitions in the optical domain, i.e., a quadrupole transition at 435 nm and an octapole transition at 467 nm with natural line width of 3 Hz and 3 nHz respectively^18,19^. Stabilizing of clock laser’s frequency, for probing such ultra-narrow transitions, requires tremendous effort for generating ultra-stable and ultra-narrow linewidth frequency using ultra low expansion (ULE) cavity^1,20–22^ and fast servo controller. Atomic transitions used for the production of ytterbium ions through photoionization, laser cooling of ions and repumping of the metastable states of the ions, have typical natural linewidth of tens of MHz and therefore the frequencies of those lasers need to be stabilized within same range, i.e., few tens of MHz for the above purposes^5^.

The present article reports about simultaneous and long terms frequency stabilization of four different lasers, used for production and laser cooling of ytterbium ions, through a wavelength meter. In order to measure the absolute frequencies of the laser, the wavelength meter is calibrated with respect to a Doppler broadening free absorption peak of Rb atoms^23^. Doppler broadening free absorption spectra of Rb atoms are recorded through saturated absorption spectroscopy (SAS) through a Rb vapour cell kept at room temperature. One of the key advantage of wavelength meter based laser frequency stabilization technique is that it allows simultaneous locking of multiple number of lasers with wide wavelength region.

## Experimental setup

**Figure 1:**
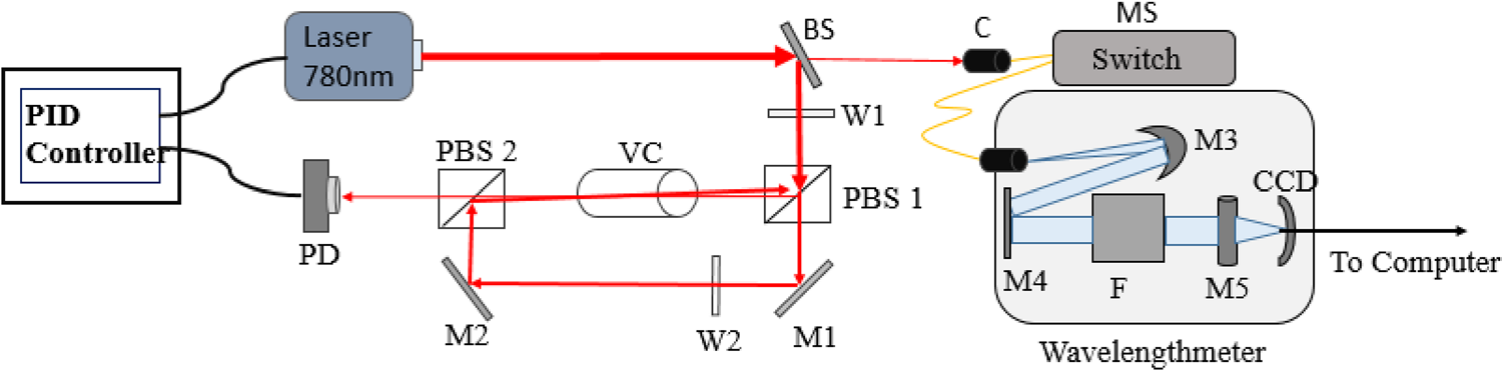
Schematic representation of the experimental setup for laser locking using saturated absorption spectroscopy (SAS). Here, BS—90:10 beam splitter, W1, and W2—quarter-wave plates, PBS1 and PBS2—polarizing beam splitters, VC—Rb vapour cell, M1, M2, M3 and M4—mirrors, M5—cylindrical mirror, PD—photodiode, MS—multichannel switch, C—fibre coupler, F—Fizeau interferometer, CCD—charged coupled devices.

A wavelength meter (WS-7, Highfiness) along with a multichannel switch have been used in the experiment for measuring as well as stabilizing laser frequencies. This wavelength meter consists of a fiber-coupled optical unit based on Fizeau interferometer that create interference patterns and capable of covering a wide range of wavelength i.e., 350–1120 nm. Interference patterns generated within the wavelength meter are then detected by two charge-coupled device (CCD) arrays and laser frequencies are estimated, with an absolute measurement accuracy of 60 MHz, in comparison with a stored internal reference pattern. Initially, a built-in neon lamp in the wavelengthmeter is used to calibrate the wavelength meter and a multichannel fiber-switch is used for simultaneous measurement of multiple laser output frequencies. A proportional-integral-derivative (PID) regulator’s analog output is used for simultaneous frequency locking for all these lasers. Neon lamp cannot be used continuously as a reference for frequency calibration because it has limited shelf life and the wavelength meter is recalibrated in a regular interval of time. But during this calibration period the wavelength meter stops measuring wavelengths for certain time. In order to achieve highest possible accuracy and long term stability in the frequency measurement, the wavelength meter needs to be recalibrated in regular intervals of time and eventually introduces error in absolute frequency measurement during recalibration period. To overcome this shortcoming the wavelength meter has been calibrated and stabilized by a continuous and stable reference, so that it can be used uninterruptedly for a longer period of time avoiding multiple times recalibration. In our experiment we have calibrated the wavelength meter to a transition frequency of atomic Rubidium using Doppler-free SAS^23–27^ technique. In the present experiments, an atomic transition of ^85^Rb with absolute wavelength 780.24392 nm has been used as the reference for calibrating the wavelength meter and the schematic diagram of the experimental setup has been presented in Fig. 1. A tuneable extended cavity diode laser (ECDL), operating at ~ 780 nm, is used for recording absorption spectra of Rb atoms. Light from the laser is coupled to the wavelength meter through a single mode optical fibre and then collimated by a mirror, before entering the solid-state Fizeau-interferometers. As shown in Fig. 1, laser output is first split into two parts using a 90:10 beam splitter and the weaker part of the laser beam is carried to the wavelength meter through the multichannel switch. The stronger part of the laser is used for the experiment i.e., for recording Doppler broadening free absorption spectra of Rb atoms. The laser beam passes through a quarter wave ( λ/4) plate followed by a polarizing beam splitter (PBS) and further splits the beam into two parts i.e., the pump and probe beams. The optical axis of the quarter wave plates is rotated in such a way that one of the beam becomes a high intense pump beam (~ 1 mW) and passes through the Rb vapour cell. The weaker part of the beam (~ 0.1 mW) is used as probe beam and passes through the Rb vapour cell from the other end i.e., in counter-propagating direction to the pump beam. The SAS spectra is recorded by detecting the probe beam on a photodiode (APD430A/M, Thorlabs) and then the signal is connected to a PID controller (DigiLock 110, Toptica) and the laser is locked to on one of the atomic transitions of Rb and this locked laser signal is used as the reference signal for the wavelength meter.

## Results and discussion

Laser locking is a useful technique for stabilizing laser’s output frequency with respect to a reference transition frequency^28^ and very commonly used in the precision experiments. Locking a laser to a particular reference peak requires a feedback control in terms of an error signal, which is proportional to the offset or detuning of the frequency from its desired value, i.e., the reference frequency. The error signal is fed back to the laser controller for tuning its output frequency.

**Figure 2:**
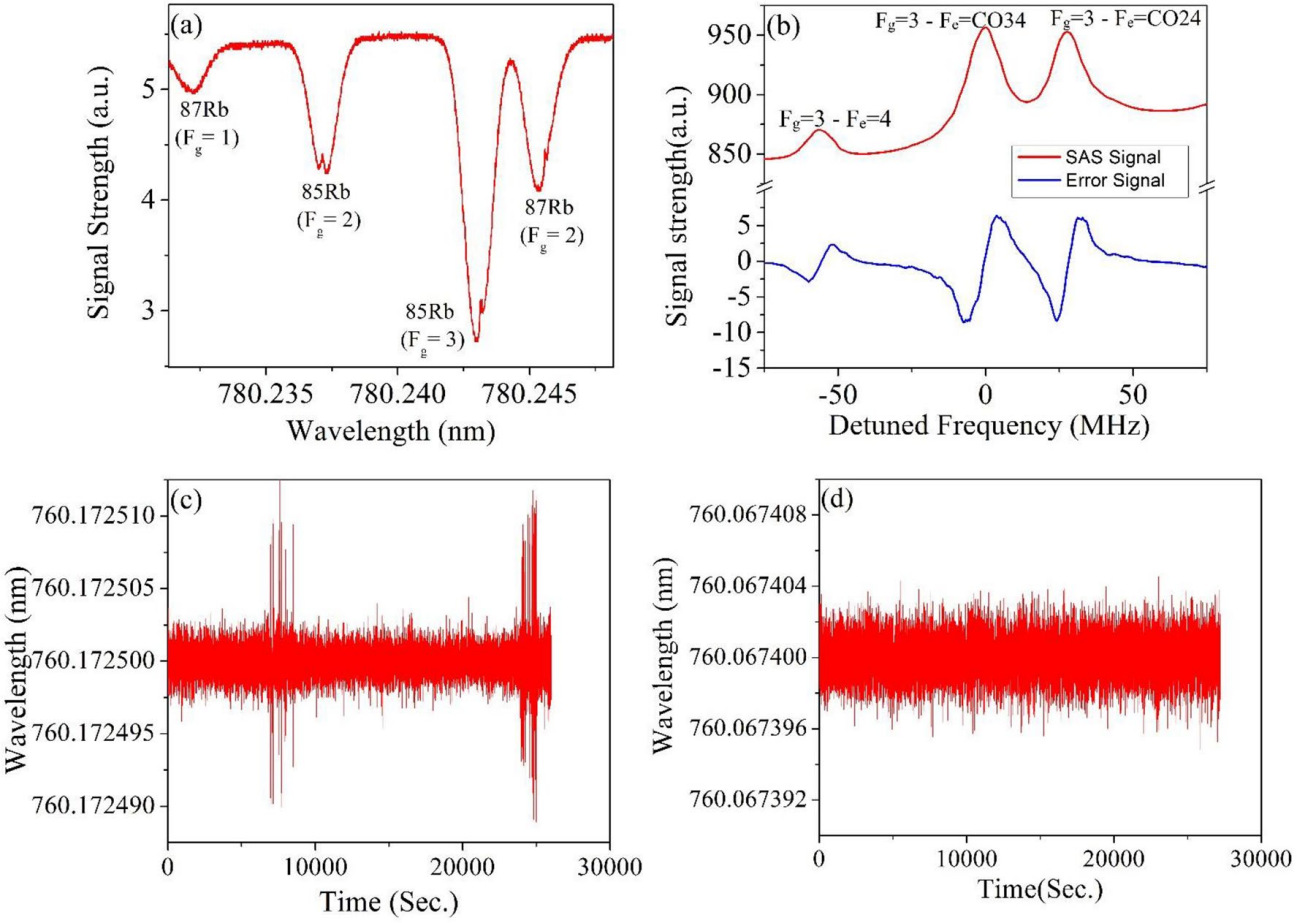
(**a**) Doppler broadening free saturated absorption spectra (SAS) for D_2_ transition of ^87^Rb and ^85^Rb atoms (y-axis-signal strength (a.u.). (**b**) Doppler broadening free SAS peaks (red line) for hyperfine transitions F_g_ = 3 to F_e_ = 4, F_g_ = 3 to F_e_ = CO34 (Crossover peak of F_e_ = 3 and F_e_ = 4) and F_g_ = 3 to F_e_ = CO24 (Crossover peak of F_e_ = 2 and F_e_ = 4) of ^85^Rb and their first order derivative as error signal (blue), the peak corresponding F_g_ = 3 to F_e_ = CO34 transition (υ = 384.229149 THz) has been used as the reference signal for calibrating and locking the wavelength meter. (**c**) Wavelength variation of a frequency stabilized laser, while the wavelength meter uses inbuilt Neon lamp as the reference of calibration. (**d**) Wavelength variation of a frequency stabilized laser, while the wavelength meter is calibrated to the SAS peak of ^85^Rb.

When a laser beam passes through an atomic vapour cell and its frequency is scanned around resonance absorption frequencies, specific absorption pattern is observed due to laser-atom interaction. But due to thermal motions of the atoms within the vapour cell, Doppler broadening occurs and results into frequency broadened absorption spectra, much broader than the natural linewidth. Let’s consider a group of atoms having linear velocity component *v*_*x*_ along the propagation direction of the counter propagating laser beams, i.e., pump and probe beams. When the pump and probe beams interact with those group of atoms, resonant absorption frequency will be shifted due to Doppler Effect and the Doppler shift depends on the relative velocity of those atoms with respect to the laser beams, can be expressed as

$$\nu = \left( {1 \pm \frac{{{\varvec{v}}_{{\varvec{x}}} }}{c}} \right)\nu_{0 }$$

where *ν*_0_ is the resonance frequency for the atoms are in rest, i.e., atoms have no velocity component along the propagation direction of the laser beams, c is the velocity of light, and *ν* is the Doppler-shifted resonance frequency. In order to minimize the Doppler broadening of absorption spectra, saturated absorption spectroscopy (SAS) technique is used where counter-propagating high intense laser beam, named as pump beam and low intense probe beam passes through the vapour cell in counter propagating configuration. The linewidth of these saturated absorption spectra is much narrower than the Doppler broaden absorption spectra. Width of the Doppler broadening free absorption spectra, i.e., saturated absorption spectra, depends on the natural linewidth of that atomic transition. Width of the SAS spectra also depends on the power of the incident laser beam and broadened linearly with the increase of laser power. Hence, the linewidth of the SAS spectra can also be optimized by reducing the power of the laser beam. Usually Doppler broaden absorption spectra have linewidth of hundreds of MHz but for SAS spectra linewidths are achieved within tens of MHz. Thus, output of any laser can be stabilized or locked to the peak of a reference SAS signal. In the present study Rb vapour cell has been chosen for generating SAS signal as Rb exists in vapour state at room temperature and no additional heating is required. In addition, atomic Rb provides many closely spaced transition frequency in the optical domain and eventually provide multiple number of Doppler broadening free SAS^23,29–31^ transitions frequencies over a wide wavelength range. Natural Rb atoms have two isotopes with relative abundance of 72% for ^85^Rb and 28% for ^87^Rb. In the present experimental setup, the D_2_ hyperfine transitions (5S_1/2_–5P_3/2_) of Rb atoms at ~ 780 nm have been probed for obtaining Doppler broadening free SAS peaks. Figure 2a depicts the absorption spectra with the SAS peaks for ^87^Rb (F_g_ = 1, 2) and ^85^Rb (F_g_ = 2, 3). In our experiment we are using ^85^Rb which have hyperfine transitions F_g_ = 3 to F_e_ = 4, F_g_ = 3 to F_e_ = CO34 (Crossover peak of F_e_ = 3 and F_e_ = 4) and F_g_ = 3 to F_e_ = CO24 (Crossover peak of F_e_ = 2 and F_e_ = 4) with corresponding linewidths of 13.73 MHz, 20.75 MHz and 21.83 MHz respectively. Our measured linewidths as mentioned in Table 1 are in good agreement with earlier measured and reported values^29,32^. One of the modulated SAS peak of ^85^Rb, corresponding to the transition F_g_ = 3 to F_e_ = CO34 at 780.24392 nm (shown in Fig. 2b) has been utilized for locking the laser frequency through a feedback controller.

**Table 1:** Details of the hyperfine transitions observed and their respective linewidth.

Peak	Hyperfine transition	FWHM (MHz)
1	F_g_ = 3 to F_e_ = 4	13.73
2	F_g_ = 3 to F_e_ = CO34	20.75
3	F_g_ = 3 to F_e_ = CO24	21.83

Figure 2b depicts the SAS peaks and their first-order differential signal generated by the PID controller and this differential signal is used as the corresponding error signal for locking the laser to a specific SAS peak. The differential signal corresponding to a SAS peak represents the zero point in the error signal and both side of the zero points have opposite sign in the error signal. In this error signal (with modulation frequency of 3.97 kHz and amplitude 0.119 V_PP_), the negative and the positive slopes define the lowest and highest point of the locking frequencies and the zero point of the error signal has been used as the reference for calibration of the wavelength meter. In order to demonstrate the advantages or benefits of calibrating the wavelength meter to a reference SAS peak in comparison to inbuilt neon lamp, frequency stabilized laser frequency have been recorded over a very long period by calibrating the wavelength meter with the neon lamp and SAS peak of atomic Rb respectively.

Figure 2c demonstrate the frequency stability of the measured output of the laser operating at ~ 760 nm and locked to the wavelength meter, while the wavelength meter is calibrated with the inbuilt neon lamp. As it is evident from Fig. 2c that the wavelength meter needs recalibration in short time to ensure that the lasers remain locked at the desired value, as the neon lamp is not in on state continuously. During the recalibration period the measurement of wavelengthmeter stop and the laser starts drifting from its operating frequency. The output frequency shows huge fluctuation during the period of its calibration and it happens in regular interval, which is quite disturbing and not acceptable for many of the precision experiments like trapping of atoms through magneto optic traps (MOT) or laser cooling of atoms and atomic ions.

The above discussed issue regarding recalibrating the wavelength meter with the neon lamp at regular interval can be solved by using a SAS peak of Rb atoms as the reference of calibration instead of the neon lamp. As the reference SAS peaks of Rb atoms are highly stable and insensitive to atmospheric perturbation, it is capable of locking the laser outputs very efficiently over a very long time. The SAS peak is used as a continuous source for calibration and doesn’t require multiple times recalibration of the wavelength meter. Figure 2c,d depict the wavelength drift and its fluctuations of a frequency stabilized laser’s output, while the wavelength meter is calibrated with the inbuilt neon lamp and the SAS peak of the Rb atoms respectively. It has been observed that calibrating the wavelength meter with the SAS peak of the Rb atoms is quite advantageous as it give very good long term stability.

**Figure 3:**
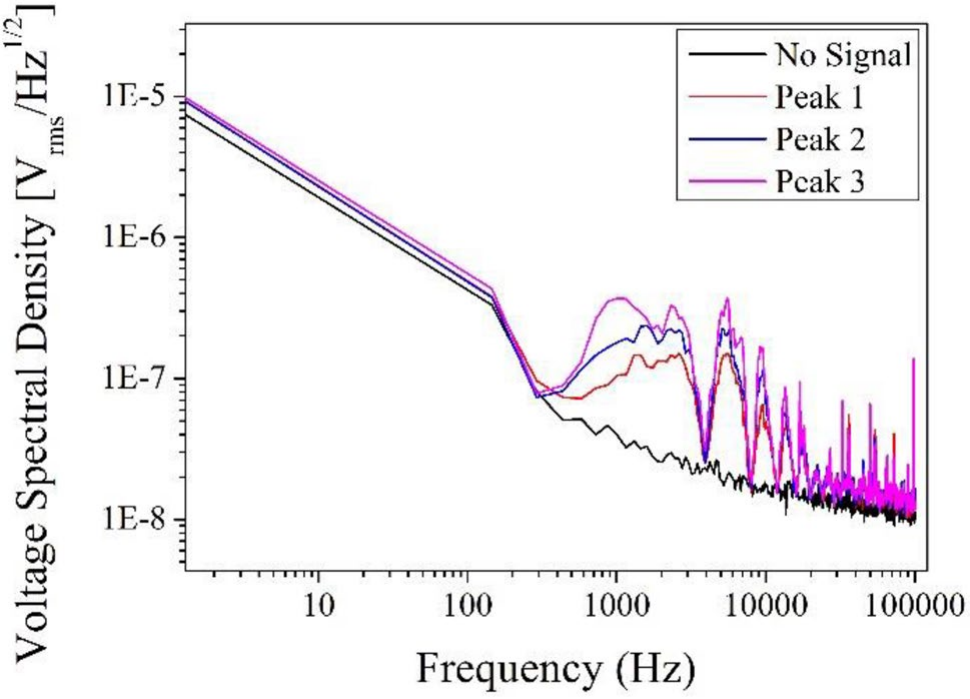
Voltage spectral density of noise floor of the spectrum analyser (black) and error signals generated through the SAS peaks corresponding to the hyperfine transitions F_g_ = 3 to F_e_ = 4 (Peak 1—red), F_g_ = 3 to F_e_ = CO34 (Peak 2—blue) and F_g_ = 3 to F_e_ = CO24 (Peak 3—purple).

In order to estimate the short-term stability of the locked laser’s output frequency, the Fast Fourier Transformation (FFT) have been performed for the error signals corresponding to different SAS peaks of Rb atoms. The voltage spectral density of noise floors of the spectrum analyser along with the SAS peaks has been presented in Fig. 3. The respective noise density of the SAS peaks which have hyperfine transitions F_g_ = 3 to F_e_ = CO24 (Crossover peak of F_e_ = 2 and F_e_ = 4), F_g_ = 3 to F_e_ = CO34 (Crossover peak of F_e_ = 3 and F_e_ = 4) and F_g_ = 3 to F_e_ = 4 are estimated to be 0.23 μv/Hz^1/2^, 0.21 μv/Hz^1/2^and 0.14 μv/Hz^1/2^@2 kHz respectively, as presented in Fig. 3.

**Figure 4:**
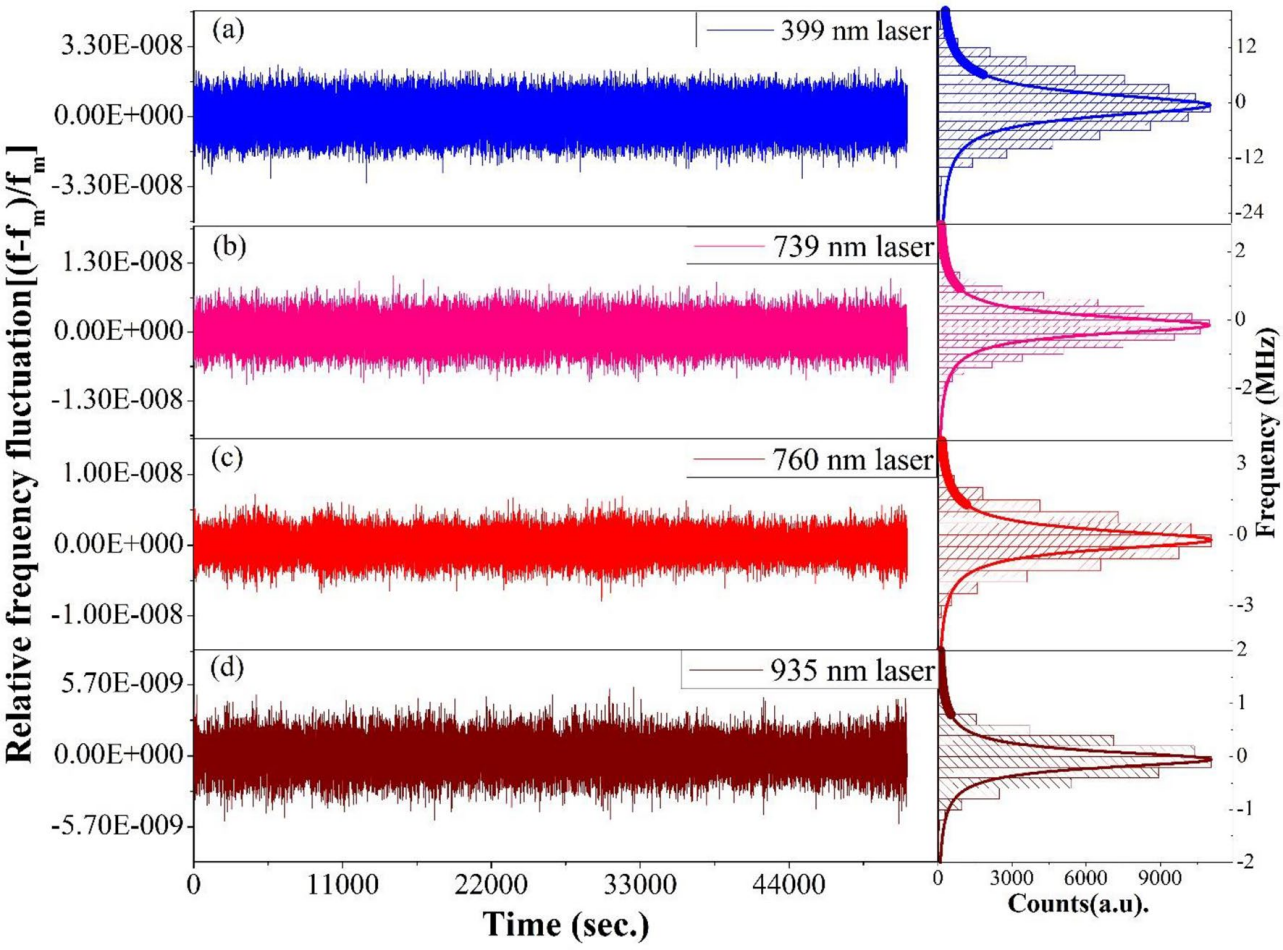
Relative frequency fluctuations of the frequency stabilized lasers operating at ~ (**a**) 399 nm, (**b**) 739 nm, (**c**) 760 nm and (**d**) 935 nm respectively. Corresponding histograms (Gaussian) show distribution of frequency offsets with respect to the central frequencies for each lasers.

After calibrating and stabilizing the wavelength meter with the SAS peak of the Rb atoms, four lasers operating at different wavelengths have been locked using the PID controller of the wavelength meter. Operational wavelengths of those four laser are ~ 399 nm 369 nm (generated from second harmonic generated signal of 739 nm laser), 935 nm and 760 nm and are used in an experiment for the production and laser cooling of ^171^Yb^+^ ions. Through the calibrated wavelength meter all four lasers were locked simultaneously to their respective wavelength over a period of 15 h. Figure 4 shows drift of wavelengths and their fluctuations for all those four frequencies stabilized lasers, the corresponding histograms in the right hand side shows the distribution of deviations of the laser’s output frequency from their central frequencies with FWHM 15.02 MHz for 399 nm, 1.46 MHz for 739 nm laser, 2.42 MHz for 760 nm laser and 0.87 MHz for 935 nm laser. Above results show that the stability as well as the linewidth of the four different laser stay well within the required limit for using them efficiently in the experiment of production and laser cooling of ^171^Yb^+^ ion within a radio frequency ion trap.

## Conclusion

A simple and effective method for frequency stabilization of multiple lasers using a wavelength meter has been presented. The wavelength meter is calibrated to a Doppler-broadening free D_2_ atomic transition of ^85^Rb through saturated absorption spectroscopy, instead of calibrating it to the in-build neon lamp. SAS peak of the atomic rubidium as the external reference enables the wavelength meter for uninterrupted measurement over very long period of time and consequently long term frequency stability achieved for the laser outputs. Four lasers of different wavelengths have been frequency stabilized through the wavelength meter for more than ten hours and all of them provide excellent frequency stability with a drift of 0.013(8) kHz/h estimated by measuring the shift of the mean frequency of the laser over a long period of time and line widths of 15.02 MHz for 399 nm, 1.46 MHz for 739 nm laser, 2.42 MHz for 760 nm laser and 0.87 MHz for 935 nm laser.

## Supplementary Information

Supplementary Information.

## Data Availability

The datasets generated and analysed during the current study are available at https://data.mendeley.com/datasets/9d3zxwyztm.
