# Supplementary material for: Frequency stabilization of multiple lasers to a reference atomic transition of Rb
Source: Sci Rep. 2022 Nov 30;12:20624. doi: 10.1038/s41598-022-24952-6 (PMC9712644; doi:10.1038/s41598-022-24952-6)
Supplement: Supplementary file 1 — Supplementary Information. [file 41598_2022_24952_MOESM1_ESM.docx]

# Datasets for the article “Frequency stabilization of multiple lasers to a reference atomic transition of Rb”

The datasets generated and analysed during the current study are available in the Mendeley data. Link for the data: - <https://data.mendeley.com/datasets/9d3zxwyztm>
